# Turning poop into profit: Cost-effectiveness and soil transmitted helminth infection risk associated with human excreta reuse in Vietnam

**DOI:** 10.1371/journal.pntd.0006088

**Published:** 2017-11-27

**Authors:** Ngan Tran-Thi, Rachel J. Lowe, Janna M. Schurer, Tu Vu-Van, Lauren E. MacDonald, Phuc Pham-Duc

**Affiliations:** 1 Center for Public Health and Ecosystem Research, Hanoi University of Public Health, North Tu Liem, Hanoi, Vietnam; 2 Department of Veterinary Microbiology, University of Saskatchewan, Saskatoon, Saskatchewan, Canada; University of Newcastle, UNITED KINGDOM

## Abstract

Human excreta is a low cost source of nutrients vital to plant growth, but also a source of pathogens transmissible to people and animals. We investigated the cost-savings and infection risk of soil transmitted helminths (STHs) in four scenarios where farmers used either inorganic fertilizer or fresh/composted human excreta supplemented by inorganic fertilizer to meet the nutrient requirements of rice paddies in the Red River Delta, Vietnam. Our study included two main components: 1) a risk estimate of STH infection for farmers who handle fresh excreta, determined by systematic review and meta-analysis; and 2) a cost estimate of fertilizing rice paddies, determined by nutrient assessment of excreta, a retailer survey of inorganic fertilizer costs, and a literature review to identify region-specific inputs. Our findings suggest that farmers who reuse fresh excreta are 1.24 (95% CI: 1.13–1.37, p-value<0.001) times more likely to be infected with any STH than those who do not handle excreta or who compost appropriately, and that risk varies by STH type (*Ascaris lumbricoides* RR = 1.17, 95% CI = 0.87–1.58, p-value = 0.29; Hookworm RR = 1.02, 95% CI = 0.50–2.06, p-value = 0.96; *Trichuris trichiura* RR = 1.38, 95% CI = 0.79–2.42, p-value = 0.26). Average cost-savings were highest for farmers using fresh excreta (847,000 VND) followed by those who composted for 6 months as recommended by the WHO (312,000 VND) and those who composted for a shorter time (5 months) with lime supplementation (37,000 VND/yr); however, this study did not assess healthcare costs of treating acute or chronic STH infections in the target group. Our study provides evidence that farmers in the Red River Delta are able to use a renewable and locally available resource to their economic advantage, while minimizing the risk of STH infection.

## Author summary

Each year, hundreds of millions of people worldwide are infected with intestinal worms spread by contaminated soil, also known as soil transmitted helminths (STHs). These worms are most common in tropical climates in areas lacking good hygiene and sanitation, and negatively impact child development, quality of life, and economic wellbeing. Reuse of human excreta for fertilizer is a common practice in many low to middle income countries because farmers require a low cost source of nutrients to grow food crops eaten by people and animals. Excreta can contain microbes, such as STHs, that cause disease in people; however, composting is a known method of killing STHs. Therefore, our goal was to determine if Vietnamese rice farmers involved in this practice are at higher risk of STH infection, and to calculate the amount of money saved by farmers composting for different lengths of time, and supplementing with various commercial fertilizers. We suggest that farmers compost excreta for six months to reduce disease exposure and optimize household savings. Optimizing practices to improve food production and protect farmer health is critical for poverty alleviation in low to middle income countries.

## Introduction

Application of human excreta onto rice paddies as fertilizer is a common practice in northern Vietnam, where many farmers use single or double vault latrines, lack access to wastewater infrastructure, and have variable access to commercial inorganic fertilizers [1]. Using organic waste to fertilize fields has clear benefits for crop yield [2]; however, this practice increases certain health risks for farmers and consumers, such as infection by soil transmitted helminths (STHs)[3,4]. The STH group includes *Ascaris lumbricoides*, *Trichuris trichiura*, and hookworm spp., which are intestinal parasites that spread between people when sanitation is inadequate or when good hygiene is not practiced [4]. People are infected when they accidentally ingest infective eggs or when their skin contacts infective larvae in contaminated soil. These parasites are particularly prevalent in regions with warm, moist climates, and are included in the category of tropical neglected diseases associated with poverty.

World Health Organization (WHO) guidelines recommend that farmers compost human excreta for six months prior to application in order to inactivate STH eggs and larvae, and thereby reduce spread between people [5]. This practice is not feasible for all Vietnamese farmers, in particular those who harvest multiple crops per year or have single vault latrines that lack a chamber for long-term excreta storage. Current evidence suggests that only one-third of farmers who use human excreta follow the six-month recommendation [6], and that STH infection remains an occupational hazard associated with handling human excreta [3]. It is common practice for household members to add a handful of kitchen ash after using a latrine, as this reduces smell. A recent study characterizing *A*. *lumbricoides* egg die-off during excreta composting suggests that adding lime reliably accelerates egg inactivation so that WHO criteria for safe handling (<1 viable egg/g total solids) are met by 153 days [7]. Ascarid eggs can survive longer periods in adverse environmental conditions than other STHs, and for that reason we chose *A*. *lumbricoides* die-off as a proxy for overall STH die-off [8].

Rice farmers in some agricultural regions of Vietnam have shifted their source of fertilizer from human excreta to commercial inorganic products, either wholly or in part. It is unclear whether this trend will become universal as not all farmers are able to afford or access commercial fertilizer, and others consider human excreta a superior source of long-term nutrition for plants and soil [9]. Inorganic fertilizers are primarily imported, and their costs are influenced by a wide range of factors, including energy prices [10]. Using human waste to fertilize crops is recognized as a way to decrease household expenditures; however, it is unclear how costs and health risks associated with STH infection interact. The goal of this study was to compare the costs and STH risk associated with fertilizing rice paddies in the Red River Delta (RRD).

## Methods

### Study context

The RRD encompasses eight provinces and two major urban municipalities (Hanoi and Haiphong) in northern Vietnam. The RRD is an agriculturally intense area that produces approximately 15% of the national annual rice output [11]. Throughout the region, farmers use various combinations of human excreta, inorganic fertilizers, and animal manure to replenish soil nutrients and maximize rice yield. To generate cost estimates, we chose four fertilization scenarios: (A) Fresh human excreta (≤ 139 day storage without lime); (B) Composted human excreta (153 day storage with 10% lime as per [7]); (C) Composted human excreta (181 day storage without lime; WHO standard [5]); (D) Inorganic fertilizer. Although three scenarios (A-C) involved human excreta, we assumed that only farmers who handled fresh excreta (A) would experience STH infection risk, as the composting scenarios (B and C) met WHO standards for helminth inactivation. Risk of STH infection for Vietnamese farmers handling fresh excreta was evaluated by systematic review and meta-analysis. Our economic analysis of the four scenarios included the direct costs incurred for composting human excreta (i.e. lime) and supplementing excreta with inorganic fertilizers. Capital costs (i.e. cost to build a double vault latrine) were not included because differences in factors such as materials and design cause costs to vary substantially in the RRD, and would add a high level of uncertainty to our analysis. To estimate the direct costs, we determined nutrient content of organic fertilizer scenarios, conducted a retailer survey of inorganic fertilizers in the study area, and collected economic inputs from published sources specific to the RRD (e.g. household size, excreta production per household, annual harvest frequency, average paddy size).

### STH risk estimate

#### Systematic review

Two systematic review research questions were defined as: (1) “is there risk of infection with any STH type among farmers who work with fresh human excreta to fertilize rice paddies in the RRD, Vietnam”; and (2) “is there risk of infection with STHs, by type of helminth, among farmers who work with fresh human excreta to fertilize rice paddies in the RRD, Vietnam”. Two authors (RL and JS) used three search engines (PubMed, Embase, and ProQuest) to search titles/abstracts of English language peer-reviewed publications using the following terms: Vietnam AND ((human excreta) OR (human waste) OR (night soil) OR (fertilizer) OR (manure)). Inclusion criteria for relevance screening were: time period (2006–2016), location (Vietnam), diagnostics (stool sample tested for STH), occupation (farming), exposure (contact with human excreta), and risk assessment reporting (risk estimate and measurement of error). The Vietnamese government, in collaboration with international aid organizations, has conducted targeted STH elimination campaigns in past decades, and we restricted the time period to avoid overestimating risk. One author (NTT) conducted similar searches in two databases managed by the National Agency for Science and Technology Information to identify Vietnamese language publications, projects, theses, and government reports. Authors initially read all titles/abstracts of the search results and then full publications of the subset that appeared to meet the inclusion criteria. As well, citation lists in relevant English and Vietnamese publications were manually searched for additional papers of interest. Duplicate reports (identified by title and primary author) were removed, and all publications that met the inclusion criteria were retained and independently assessed for quality by RL and JMS using a version of the Newcastle-Ottawa scale modified from [12,13] (Table 1). Quality assessment of relevant studies evaluated the materials and methods section of each report for complete descriptions and appropriate study design, sampling methods, control of confounders, exposure and outcome assessment, inclusion and exclusion criteria, and statistical analysis. Data were extracted on author, language, region, study design, population, exposure, outcome, sample size, measure(s) of association, 95% confidence intervals, p-values, standard errors, confounders adjusted for, and number of: exposed, unexposed, outcome positive and outcome negative, from each publication and recorded in a Microsoft Excel (2013) database.

**Table 1:** Quality assessment tool used to evaluate individual publications that met inclusion criteria for reporting STH risk in farmers handling human excreta (modified from [12,13]).

Study Component	Reviewer’s Comments	Score
Selection: (≤ 6 stars)		
Representativeness of the sample:		
a) Truly representative of the average ‘__’ in the target population (all subjects or random sampling).**		
b) Somewhat representative of the average ‘__’ in the target population (non-random sampling).*		
c) Selected group of users (e.g. farmers, nurses).		
d) No description of the sampling strategy.		
Sample size:		
a) Justified and satisfactory.*		
b) Not justified.		
Non-respondents:		
a) The response rate is reported and satisfactory.*		
b) The response rate is not reported, or unsatisfactory.		
Assessment of the exposure (risk factor):		
a) Validated measurement tool. **		
b) Non-validated measurement tool, but the tool is available or described.*		
c) No description of the measurement tool.		
Comparability: (≤ 2 stars)		
The subjects in different outcome groups are comparable, based on the study design or analysis. Confounding factors are controlled.		
a) The study justifies inclusion of variables (e.g. stepwise regression), controls for confounders or effect modifiers, and has comparable outcome groups.**		
b) The study satisfied 2 of 3 above listed criteria.*		
c) The study does not provide sufficient detail to assess comparability of outcome groups.		
Outcome: (≤ 5 stars)		
Sample collection:		
a) >1 stool samples collected per respondent within one week period.*		
b) One sample collected per respondent.		
c) No description.		
Diagnostic test:		
a) >1 validated test used or 2+ blinded technicians used the same validated test.**		
b) One validated test used by a trained technician.*		
c) Diagnostic test not described, not validated, or known to misrepresent true infection prevalence.		
Statistical test:		
a) The statistical test used to analyze the data is clearly described and appropriate, and the measurement of association is reported with estimates of error (confidence interval and p-value). **		
b) The statistical test used to analyze the data is clearly described and appropriate, and the measurement of association is reported with a confidence interval or p-value.*		
c) The statistical test is not appropriate, not described, or incomplete.		
**Total Score (≤ 13 stars):**		

*—indicates 1 point

**—indicates 2 points

#### Meta-analysis

The risk of STH infection associated with handling fresh human excreta was assessed in random-effects meta-analysis using data from relevant studies that met inclusion criteria collected in the systematic review. Unadjusted raw data for disease positive, disease negative, exposed and unexposed were extracted from each manuscript and used to generate pooled relative risks (RRs) for each research question aspoor reporting of necessary sample sizes, p-values, or standard errors precluded inclusion of adjusted risk estimates in the analysis. One study [14] did not report sample size by exposure or outcome, and therefore could not be included in the meta-analysis. Statistical analysis of heterogeneity was assessed by the *I*^*2*^ statistic, where an *I*^*2*^ value above 25% denoted significant heterogeneity. Where possible, sensitivity analysis was used to assess the extent to which one or more factors, such as study, might explain observed heterogeneity. All analyses were conducted using STATA 14.

### Cost estimates

#### Nutrient standardization of organic fertilization scenarios

The Vietnamese Rice Knowledge Bank provides recommendations for nitrogen: phosphorus: potassium (NPK) amounts needed to replace soil nutrients per rice harvest [15]. To ensure that the NPK content of each organic fertilizer scenario (A-C) was standardized within the upper and lower bounds of these recommendations, we composted human excreta to monitor changes over the defined composting period. Excreta were stored in concrete vaults meant to simulate double vault latrines, and three samples per vault were analysed every 28 days over a 139 day period. Changes in moisture content (and thereby excreta weight) were also assessed to determine NPK concentrations per kg of excreta. Details of storage conditions, sample collection, and moisture assessment are reported elsewhere [7].

Total nitrogen concentration of composted excreta was assessed by the Kjeldahl method [16]. Total phosphorus content was assessed by mixing 0.5 grams of excreta to 10 mL of 98% sulfuric acid in a dried Kjeldahl flask, and pipetting 1 mL of the mixture into a 50 mL flask where it was combined with 2 mL of 2.5% ammonium molybdate and 1 mL of ascorbic acid. The solution was diluted with 40 mL dH_2_O, heated in a sand bath to 300°C for 10 minutes, cooled, and then measured at 710 nm by ultraviolet–visible spectrometer (Model U-2900, Hitachi High Technologies Corporation, Toyko, Japan). The total potassium content was determined by mixing 0.5 grams of excreta with 10 mL of 98% sulfuric acid in a dried Kjeldahl flask, and measuring the solution by atomic absorption spectrophotometer (Model AA-6800, Shimadzu Corporation, Kyoto, Japan) as per government standard [17].

To generate mean nutrient content for fresh excreta (A), we averaged the NPK and moisture content for excreta sampled over 139 days. For the compost scenarios (B,C), we conducted a simple linear regression to identify significant changes over time in NPK and moisture levels for excreta stored with 10% lime (B), and without lime (C). If no significant changes were evident, we used the last recorded estimates (i.e. 139 days). When NPK and moisture content differed significantly over time, general linear regression was used to predict levels at 153 days (B) and 181 days (C). Using these data, final NPK concentrations were calculated and compared with upper and lower NPK recommendations to identify a suitable application rate for rice paddies, and to inform the cost estimates of scenarios A, B, and C. If excreta did not provide sufficient N, P, or K, we assumed that farmers would supplement with nutrient specific inorganic fertilizers.

#### Inorganic fertilizer costs

To estimate the price of recommended NPK fertilizers, we recruited two resident volunteers in each of the eight provinces in RRD. Each volunteer visited at least one retailer in their commune in December 2016 and surveyed the shop owner to determine the price per kilogram for any brand (up to ten) of the recommended fertilizer types. We used the average retail price across all brands and communes to estimate cost-savings.

#### Cost estimate generation

To quantify the cost-savings available to farmers who substituted human excreta for inorganic fertilizer (A, B, C), we used the nutrient analysis along with estimates of excreta production [18] and household size [19] to determine the amount of organic NPK available to an average RRD household. We then used estimates of annual harvest frequency and average paddy size to calculate the average amount of NPK required per household annually [19]. The amount of organic NPK available was then compared to the average NPK required to determine the weight of human excreta that should be distributed onto rice paddies each year for each fertilization scenario. We assumed that organic NPK levels falling within the upper and lower bounds of recommended inorganic NPK application would lead to optimal rice yield. Finally, we used our estimate of RRD fertilizer prices to calculate the average cost incurred by farmers for each scenario (A-D). Sensitivity analysis was conducted to test upper and lower values for fertilizer prices, rice paddy size and household size, while holding all other values constant.

## Results

### STH risk estimate

#### Systematic review

**Fig 1:**
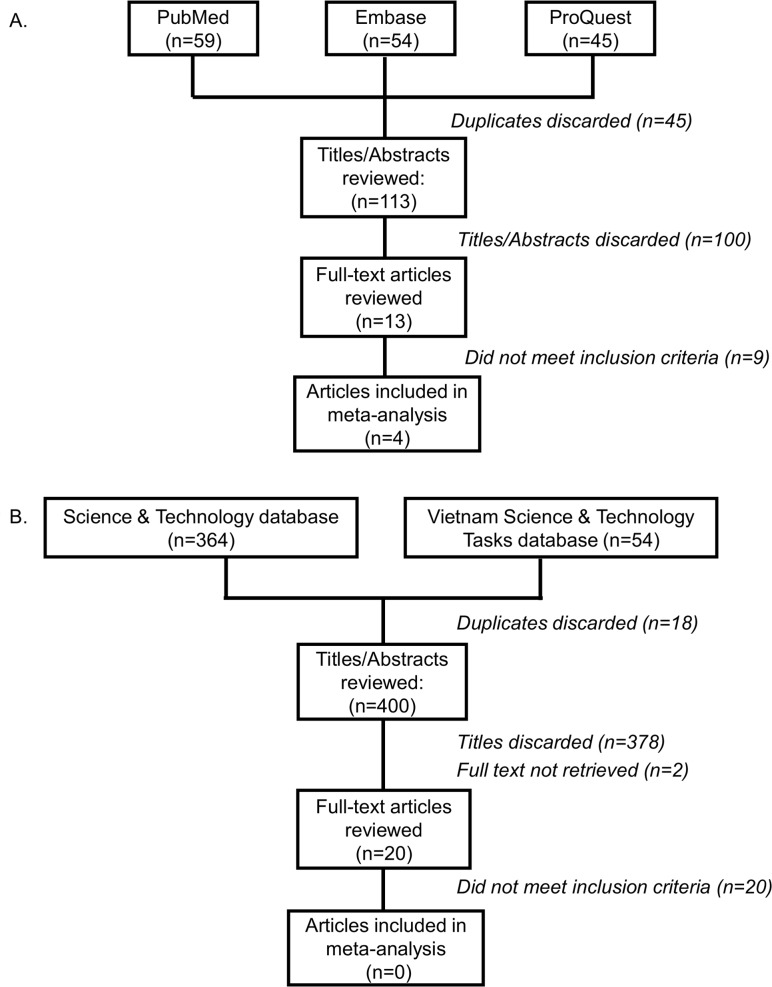
**Flow chart of search strategy and study selection for reports of STH infection following use of human excreta in Vietnam** (A) English. (B) Vietnamese.

Our search terms identified 513 unique publications for review (113 English, 400 Vietnamese; Fig 1). Of these, four English language publications and zero Vietnamese language publications, met our inclusion criteria. Overall, the quality of reporting was low to moderate, and we noted a high degree of variability in quality scores among selected publications (mean = 8, range = 6–12; Table 2). Two of four papers reported STH risk estimates in risk ratios (RR) and the other two reported odds ratios (OR).

**Table 2:** Characteristics and methodological quality of four English language, cross-sectional studies reporting the association of human excreta use in agriculture on STH infection in Vietnam (2006–2016).

Author	Region	Population Assessed	Exposure (Exposure Assessment)	Outcome (Outcome Assessment)	Sample Size	MOA (95% CI)	p-value	Controlled Confounders	Quality Score
Nguyen, PH et al. 2006 [14]	53 provinces in Vietnam	Non-pregnant women of reproductive age	(1) Using untreated feces for farming (Structured questionnaire)	(a) Hookworm (b) *A*. *lumbricoides* (c) *T*. *trichiura* (Kato-Katz technique)	N = 5127 Sample size by exposure and outcome NR.	Multivariate: OR(1,a) NR OR(1,b) = 1.3(1.0–1.6) OR(1,c) NR	NR	-Models included farming, lack of a closed latrine, zone of residence, untreated feces as fertilizer, helminth coinfection, or women’s occupation depending on helminth type model. Household data weighted by zone/commune.	6.5
Trang, DT et al. 2007 [20]	Yen So commune, peri-urban area, Hanoi, Vietnam	Farmers and their families including adults 15–70; children <72 month	(1) Use of fresh human excreta in agriculture (Household interviews)	(a) Any STH (b) Hookworm (c) *A*. *lumbricoides* (d) *T*. *trichiura* (Direct smear method)	N = 807 (E, O) +ve: (1,a) n = 77 (1,b) n = 49 (1,c) n = 40 (1,d) n = 22	Univariate RR(1,a) = 1.20 (0.93–1.55) RR(1,b) = 1.46 (1.04–2.05) RR(1,c) = 1.1 (0.76–1.58 RR(1,d) = 1.44 (0.87–2.36) Multivariate: RR(1,a) = 1.19 (0.93–1.53) RR(1,b) = 1.45 (1.03–2.03) RR(1,c) NR RR(1,d) NR	NR	- Multivariable model examined significant relationships of potential covariates including: age, gender, household hygiene practices, waste water practices, socioeconomic status, and animal husbandry for each outcome. However, which covariates were included in the final models was NR.	8
Yajima A et al. 2009 [21]	Tien Xuan commune, Hoa Binh province, Vietnam	Commune residents	(1) Use of human feces in agriculture (Questionnaire)	(a) Hookworm infection (b) *A*. *lumbricoides* (c) *T*. *trichiura* (Kato-Katz technique)	N = 101 (E, O) +ve: (1,a) n = 13 (1,b) n = 1 (1,c) n = 10	Univariate only: RR(1,a) = 0.7 (0.46–1.06) RR(1,b) = 0.21 (0.03–1.54) RR(1,c) = 0.74 (0.43–1.27)	NR	-n/a	6
Phuc PD et al. 2013 [22]	Nhat Tan and Hoang Tay communes, Hanam province, Vietnam	Commune residents >12 months of age among both individuals with primary occupation of agriculture work and individuals whose primary occupation was non-agriculture work	(1) Use of human excreta for application in field (Questionnaire)	(a) Helminth spp. (formalin-ether concentration technique) (b) *A*. *lumbricoides* (c) *T*. *trichiura* (Kato-Katz technique)	N = 1425 (E, O) +ve: (1,a) n = 381 (1,b) n = 197 (1,c) n = 330	Univariate: OR(1,a) = 1.5 (1.2–2.0) OR(1,b) = 1.4 (1.1–2.0) OR(1,c) = 1.5 (1.2–2.0) Multivariate: OR(1,a) = 1.3 (0.9–2.0) OR(1,b) = 1.3 (0.8–2.0) OR(1,c) = 1.5 (1.0–2.3)	(1,a) = 0.18 (1,b) = 0.33 (1,c) = 0.04	-Adjusted for age, gender, season	12

NR = not reported; MOA = Measure of Association

E,0 +ve = Sample size of individuals positive for both exposure (E) and outcome (O). Numbers in parentheses correspond to the combination of exposure and outcome assessed.

n/a = not applicable

OR: Odds Ratio

RR: Risk Ratio

MV: Multivariate

Quality score derived from mean score of 2 reviewers using quality assessment tool (Table 1) designed for cross-sectional studies. Maximum score of 13 points.

#### Meta-analysis

**Fig 2:**
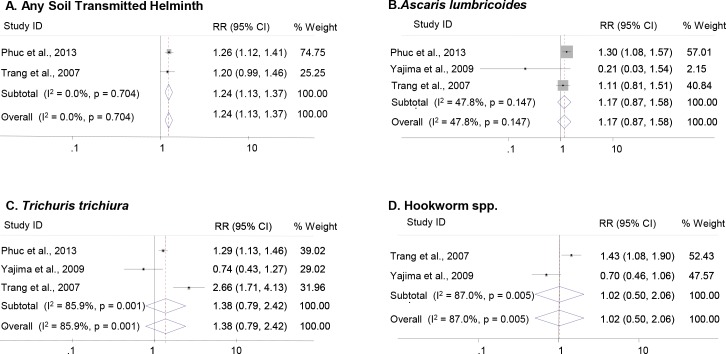
Meta-analysis forest plot describing individual and pooled RR estimates of STH infection in farmers who handle fresh human excreta in Vietnam. [A] Any Soil Transmitted Helminth [B] *Ascaris lumbricoides* [C] *Trichuris trichiura* [D] Hookworm spp. Note: Weights are from random effects analysis.

Using inverse variance weighting and random effects, our analysis found that farmers who fertilized rice paddies with fresh human excreta were 24% (RR = 1.24, 95% CI: 1.13–1.37, p-value<0.001; Fig 2) more likely to be infected with one or more STHs than farmers who did not handle fresh excreta. However, the infection risks of individual helminths were lower than the risk of any STH, and were not significant. Risk of hookworm infection (RR = 1.02, 95% CI: 0.5–2.06, p-value = 0.96) was lowest compared to other helminth types (*A*. *lumbricoides* RR = 1.17, 95% CI: 0.87–1.58, p-value = 0.29; *T*. *trichiura* RR = 1.38, 95% CI: 0.79–2.42, p-value = 0.26; Fig 2). Meta-analyses that examined infection risk with *T*. *trichiura* or hookworm demonstrated significant heterogeneity (I^2^ = 85.9% and I^2^ = 87.0%, respectively). Sensitivity analysis for the *T*. *trichiura* outcome found that removal of Yajima et al., 2009 eliminated the majority of heterogeneity [21]. The *A*. *lumbricoides* meta-analysis had also low evidence quality due to high heterogeneity among three studies (I^2^ = 47.8%); heterogeneity was eliminated by the removal of Yajima et al., 2009 (I^2^ = 0%). Sensitivity analysis could not be carried out for hookworm infection risk due to the small number of studies (n = 2) included in the meta-analysis.

### Cost estimates

#### Nutrient standardization of organic fertilization scenarios

The Vietnamese Rice Knowledge Bank recommends spreading inorganic NPK 16-16-8 or 17-12-5 fertilizers at a rate of 415–550 kg/ha for each rice harvest in the RRD [15]. These percentages are substantially higher than all NPK levels observed in organic human excreta, regardless of whether it was fresh or composted (Tables 3 and 4). On average, fresh excreta contained higher nitrogen, phosphorus, potassium, and moisture levels (1.66%, 3.23%, 2.44%, and 42.2%, respectively) than composted excreta. For excreta stored without lime (A,C), NPK levels and moisture content all decreased significantly over time (p-value<0.01 each). When excreta was composted with 10% lime (B), only nitrogen and moisture changed significantly over time (p-value <0.001 each); however, this storage method resulted in lower NPK than excreta composted without lime (C).

**Table 3:** Summary of data input sources used to assess cost and STH risk associated with four rice fertilization methods in RRD, Vietnam.

Input	Estimate	Source(s)
STH risk for farmers who handle fresh human excreta, RR (95% CI)	1.24 (95% CI: 1.13–1.37)	Systematic review and meta-analysis
NPK^1^ fertilizer recommendation for rice paddies in RRD^2^	16-16-8 or 17-12-5	[15]
Recommended NPK application rate, kg/ha per rice harvest	415–550	[15]
Recommended compost period to inactivate STH^3^ ova	153 days (with ash and 10% lime)	[7]
	181 days (with ash)	[5]
Total nitrogen in human excreta, % (A, B, C)^4^	1.66, 1.29, 1.32	Nutrient analysis
Total phosphorus in human excreta, % (A, B, C)	3.23, 1.67, 2.92	Nutrient analysis
Total potassium in human excreta, % (A, B, C)	2.44, 1.82, 2.23	Nutrient analysis
Moisture content in composted human excreta, % (A, B, C)	42.16, 9.16, 6.83	Nutrient analysis
Moisture content in newly evacuated excreta, %	93	[18]
Average excreta production, kg/person/day	0.998	[18]
Average number household members in RRD, n ± sd	Agricultural: 3.66 ± 1.38	[19]
	Not agricultural: 4.38 ± 1.19	
Average rice paddy size in RRD, ha ± sd	0.12 ± 0.096	[19]
Number of rice harvests per year	2	[19]
Retail fertilizer price in RRD, Average (min-max) ‘000’VND/kg	16-16-8: 9.2 (4–15)	Retailer survey
	17-12-5: 7.96 (3.5–12)	
	DAP: 7.7 (3–15)	
	Urea: 7.8 (4.5–12)	
Estimated retail price of lime, ‘000’VND/kg	2	Expert opinion
Average lime weight (10% of excreta), kg/year	133.33	Nutrient analysis

^1^ Nitrogen-phosphorus-potassium

^2^Red River Delta

^3^Soil transmitted helminth

^4^A = Fresh human excreta (≤ 139 day storage without lime); B = Composted human excreta (153 day storage with 10% lime; C = Composted human excreta (181 day storage without lime)

**Table 4:** Observed nutrient and moisture content of human excreta composted by two methods in a simulated double vault latrine system over 139 days with linear regression to predict values at 153 and 181 days.

	Sampling day						Average (≤139 days)	Predicted	P-value
	1	27	55	83	111	139			
Ash								(181 days)	
Total Nitrogen (%)	1.937	1.770	1.647	1.587	1.527	1.497	1.661^1^	1.317^2^	0.000
Total Phosphorus (%)	3.537	3.314	3.174	3.152	3.124	3.105	3.234^1^	2.918^2^	0.000
Total Potassium (%)	2.704	2.427	2.412	2.388	2.370	2.360	2.443^1^	2.228^2^	0.002
Moisture (%)	61.39	56.47	48.31	39.63	28.60	18.54	42.155^1^	6.830^2^	0.000
Ash + 10% Lime								(153 days)	
Total Nitrogen (%)	1.820	1.643	1.550	1.477	1.413	1.377	1.547	1.290^3^	0.000
Total Phosphorus (%)	1.838	1.696	1.628	1.614	1.608	1.604	1.665^3^	N/A	0.565
Total Potassium (%)	2.054	1.865	1.849	1.835	1.822	1.816	1.874^3^	N/A	0.477
Moisture (%)	53.40	48.81	40.38	32.83	21.43	11.96	34.802	9.163^3^	0.000

^1^Model inputs for Scenario A (≤ 139 day storage without lime)

^2^Model inputs for Scenario C (181 day storage without lime)

^3^Model inputs for Scenario B (153 day storage with 10% lime)

We compared the amount of organic NPK available per household to the annual RRD soil requirements and found excreta to be a good source of phosphorus and potassium but not nitrogen. We determined that farmers should apply fresh excreta (A) at a rate of 3459 kg/ha to meet but not exceed phosphorus and potassium requirements. At this rate, 425 kg of fresh excreta would be used to fertilize an average sized rice paddy, which is 65% of the total excreta produced by an average RRD family per year. Only 43% of the minimum nitrogen requirement would be met, indicating that farmers would need to supplement a nitrogen-based fertilizer (urea or diammonium phosphate (DAP)) for optimal rice production. Nutrient and moisture loss during composting meant that average RRD households did not produce sufficient excreta to meet soil requirements after 153 days or 181 days of storage. Excreta composted with 10% lime (B) or without lime (C) could be combined with inorganic NPK fertilizer at 6:1 or 5:1 ratios (organic: inorganic), respectively, in order to meet all nutrient requirements.

#### Inorganic fertilizer costs

Our retailer survey collected cost data for the two recommended NPK fertilizers (16-16-8 and 17-12-5) and two nitrogen-based fertilizers (urea and DAP) from 35 fertilizer retailers across eight provinces in the RRD. All retailers agreed to participate in the survey, although not all could provide prices for each fertilizer type. We obtained cost estimates for 41 NPK 16-16-8 products from 27 retailers, 25 NPK 17-12-5 products from 17 retailers, 74 urea products from 34 retailers, and 38 DAP products from 24 retailers. Among all products and brands, fertilizer prices ranged from 3,000 to 15,000 VND per kilogram (Table 3). When ranked by average price, NPK 16-16-8 was most expensive (9,000 VND/kg), followed by NPK 17-12-5, urea, and DAP (all approximately 8,000 VND/kg).

#### Cost estimate generation

**Fig 3:**
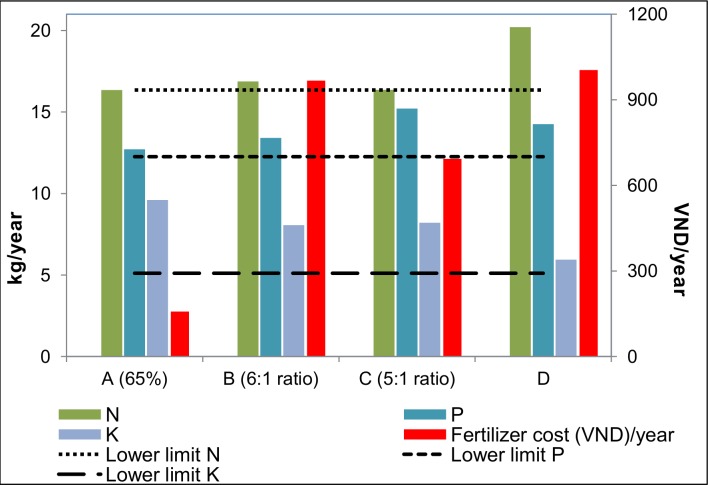
Nutrient content and annual cost of four fertilization scenarios utilizing human excreta and/or commercial fertilizer in Vietnam. Nitrogen (N), phosphorus (P), potassium (K). Scenarios: A -Fresh human excreta (≤ 139 day storage) supplemented with nitrogen; B—Composted human excreta (153 day storage with 10% lime) supplemented with NPK; (C) Composted human excreta (181 day storage) supplemented with NPK; (D) Inorganic NPK fertilizer.

Rice paddies of average size in the RRD require 118.4 kg per year of inorganic NPK 16-16-8 or 17-12-5 fertilizer. Under scenario D, farmers applying one of the recommended NPK fertilizers in this amount incur an average annual cost of 1,004,000 VND (range: 416,000–1,783,000 VND; Fig 3). Farmers using fresh excreta would need to purchase a nitrogen-based fertilizer (urea or DAP), which would cost an average of 157,000 VND/year if applied up to the minimum recommended level. This implies an annual cost savings of 847,000 VND for scenario A. Under scenario B, farmers incurred costs for lime (average: 267,000 VND/year) and NPK fertilizer (average: 701,000 VND/year), representing a household saving of 37,000 VND annually. Farmers operating under scenario C incurred costs for NPK fertilizer only (average: 693,000 VND/year), and could save on average 312,000 VND annually. According to the sensitivity analysis, costs varied according to rice paddy size, household size, and fertilizer costs; however, unless NPK fertilizer becomes significantly cheaper than the cost of lime, the fertilizer scenarios maintain their ranking with regard to cost savings.

## Discussion

This study adds to current knowledge about the opportunities and risks associated with reusing human excreta to fertilize rice plants in one region of Vietnam. Our finding that handling fresh excreta increases the risk of STH infection in farmers (RR = 1.24, 95% CI: 1.13–1.37) emphasizes the importance of adequately treating excreta to inactivate STH life stages. Furthermore, the risk is not limited to farmers as fresh excreta reuse facilitates STH spread to other commune residents, and ultimately to consumers, through food, water, and environmental transmission routes. This practice is one factor contributing to the high prevalence of *A*. *lumbricoides* (44.4%; N = 34 million), *T*. *trichiura* (23.1%; N = 17.6 million) and hookworm (28.6%; N = 21.8 million) infections in Vietnam [23]. Our study did not assess healthcare costs associated with STH prevention, treatment, or chronic disability. Individuals with low intensity infections are often asymptomatic; however, those with high intensity infections can experience a variety of acute or chronic conditions (e.g. diarrhea, abdominal discomfort, anemia and rectal prolapse) that reduce quality of life and may require costly medical interventions to treat [4]. Long-term sequelae of chronic infections, such as impaired cognitive development and growth faltering, can negatively impact lifelong earnings and contribute to the cycle of poverty in low resource communities. Although our study showed that farmers using fresh excreta benefitted from the largest cost-savings in fertilizer expenditure, the direct and indirect societal costs incurred due to prolonged STH infection would likely outweigh these savings.

Despite laws that prohibit use of human excreta for agriculture in Vietnam, this practice remains common among certain farming groups [24]. Human excreta is perceived as more valuable than animal manure due to differences in dietary protein content, and it is believed to improve soil structure more sustainably than inorganic fertilizers [9]. Although many farmers compost excreta, WHO recommendations for hygienic composting are not commonly followed, as farmers harvest multiple crops per year and are unwilling or unable to store excreta for six months prior to use [5,6]. Some misperceptions about the reasons for safe composting might influence farmer willingness to use fresh excreta. For example, focus group participants in the RRD emphasized ease of application and benefits to soil structure, rather than the benefits of composting to protect human health [1]. Our alternative to the WHO standard, composting for 153 days with 10% lime to accelerate STH inactivation, was not a reasonable alternative for farmers prioritizing cost savings. However, for farmers less concerned about cost savings, the 10% lime compost strategy could be further accelerated to 111 days by adding aeration to latrines, which would allow excreta to be safely handled at more frequent intervals [7].

Human excreta use in crop agriculture was previously estimated to represent 83 million USD in fertilizer import savings to the Vietnamese economy [6], which is one-fifth of the 2014 net expenditure on inorganic fertilizer importation (384 million USD) [25]. Our cost analysis indicated that farmers could save 37,000–847,000 VND/yr (1.48–37.28 USD, $2017) [26] by using human excreta. While these savings might appear low, they represent 1–22% of a farmer’s average annual income in the RRD [27]. Furthermore, the savings could represent a higher percent of annual income in regions that are less fertile, where rice yields are lower, or in remote locations where transportation challenges result in higher commercial fertilizer costs. Another report, suggesting that household excreta traded on the domestic market could contribute up to 15% of household income for those in the lowest income quintile, is in line with our analysis [6]. Therefore, it is unlikely that low-income rural farmers would be willing to universally replace organic fertilizers of human origin with inorganic commercial fertilizers. This was previously demonstrated by farmers who were given non-composting latrines and who ultimately broke the seals open to access the excreta [24].

Our nutrient analysis of human excreta originating from the RRD and composted over time demonstrated excreta to be an adequate organic source of phosphorus and potassium, but not nitrogen, for plant growth. Therefore, all of the scenarios using human excreta (A-C) required additional inorganic fertilizer in order to meet the recommendations for optimizing rice yield. It is not clear how far outside Vietnam these results should be extrapolated as differences in dietary intake directly influence NPK excretion, and soil supplementation requirements vary regionally. Furthermore, our analysis was based on total excreta collected in a double vault latrine, rather than waste separated into liquid and solid components, as occurs in some other regions that use excreta. However, beyond the immediate economic and agricultural gains to reusing excreta, there are global benefits to nutrient recycling. It is estimated that the demand of phosphate rock will outweigh supply by the mid-21^st^ century, which has important consequences for food security as phosphorus is essential for plant growth [28]. As access to a hygienic toilet (flush, pour flush, sulabh or double vault latrine) is still regionally variable in Vietnam (61.6–96.7% of homes containing a latrine), an opportunity currently exists to optimize nutrient recovery infrastructure in homes requiring sanitation upgrades [27].

Our systematic review and meta-analysis found a statistically significant higher risk of infection with any STH among Vietnamese farmers who use human excreta, and highlighted the limited volume of evidence to describe this association. Only four studies met our inclusion criteria, despite searching academic and grey-literature sources in Vietnamese and English. Of these, only three were included in meta-analysis due to poor reporting quality. Out of a possible score of 13, two studies achieved a quality score of 50% or lower. Our findings showed that studies often did not report descriptions of appropriate sample size determinations, confounders controlled for, sample size for positive exposure and/or outcomes, as well as risk estimates or associated p-values. However, aside from one study, all studies were from the RRD study area, included participants of similar age and gender, and estimated exposure and outcomes using similar methods. Each study had slightly different definitions for agricultural use of human excreta, and therefore our meta-analysis included both individuals whose primary occupation was rice farming, but also those who worked with human excreta in other ways aside from direct field application. Inclusion of Yajima et al., 2009 in the meta-analyses of both *T*. *trichiura* and *A*. *lumbricoides* produced significant heterogeneity in the estimates of infection risk. This study had a small sample size and very low prevalence of STHs (i.e. one case of *A*. *lumbricoides* detected), leading to low risk ratios and wide confidence intervals. The study did not provide information on approaches used to measure or control for potential confounders, further adding to the difficulty in interpretation of protective properties of human excreta use in STH. Pooled results by STH type revealed the lowest risk for hookworm infection, and significant variation in studies combined in meta-analysis for this outcome. It was not possible to examine potential factors contributing to heterogeneity in hookworm risk estimates due inclusion of only two studies in meta-analysis. Therefore, in order to better understand factors influencing infection and to substantiate the limited body of evidence on STH risk in the RRD of Vietnam, additional research employing high methodological rigour is warranted.

Although our study attempts to represent the typical situation in the RRD, much of our data comes from Ha Nam province exclusively which may differ in relevant ways from other RRD provinces. The estimate of risk was limited by the number of estimates reported in the literature and we assumed that STH risk was equal in scenarios B-C. Human excreta was collected from various households and mixed before analysis. Thus, results may not accurately reflect the nutrient and moisture content of unmixed excreta if collected and analysed from time of defecation. Costs related to the removal of excess human excreta, latrine construction and maintenance, and personal safety equipment were not explored.

## Conclusions

Our study confirmed that human excreta is a significant and sustainable source of nutrients needed for crop fertilization. Its use as agricultural fertilizer, a common practice in Vietnam, offers direct benefits to rice farmers. Human health, agricultural productivity, household earnings are optimized when farmers follow WHO standards for excreta use and government standards for crop fertilization; however current policies prohibit excreta use altogether and therefore may need to be revisited. Furthermore, our results suggest that farmers and the Vietnamese economy would benefit by forward thinking public health messaging promoting STH prevention, such as safe excreta handling strategies, personal protective equipment (e.g. gloves and boots) and regular anthelmintic prophylaxis, rather than an outright ban on excreta use. This study highlights agricultural policies needing further attention, and demonstrates the value of promoting research that provides innovative solutions for safely and economically extracting nutrients from human excreta.
